# 

**Published:** 1956-01

**Authors:** 


### THE
### MEDICAL JOURNAL
### OF THE
### SOUTH-WEST

*Incorporating*
*The Bristol Medico-Chirurgical Journal*

ARMED
FORCES
FEB 7 1956
MEDICAL
LIBRARY

*January 1956*

VOL. 71 (i). No. 259 Price 4/-

